# New Insights in Wettability and Surface Repellency of Advanced Materials

**DOI:** 10.3390/ma15238434

**Published:** 2022-11-26

**Authors:** Jie Wang, Xianghui Hou

**Affiliations:** 1 School of Materials Science and Engineering, Nanjing Institute of Technology, Nanjing 211167, China; 2 Faculty of Engineering, University of Nottingham, University Park, Nottingham NG7 2RD, UK

“New Insights in Wettability and Surface Repellency of Advanced Materials” is a new Special Issue of *Materials*, which commits to publishing original and review papers on the recent progress of wettability and surface repellency of materials, including new findings and understanding of surface repellent materials and related theory, design, fabrication, characterization, and applications.

Research topics on the wettability and surface repellency of materials have received tremendous interest in the past few decades, strongly motivated by their wide range of industrial applications due to their self-cleaning, anti-fouling, anti-soiling, antibacterial, and ice mitigation properties, among others. The attachment and accretion of undesirable liquid/solid substances, microbacteria, or even marine organisms on construction surfaces significantly pose serious operational and health/safety challenges. Various surface design strategies of advanced materials have been applied to mitigate the impacts of unfavourable substance accretion, and different levels of success have been achieved [1,2].

It is normally believed that when a liquid contacts a solid surface, the wettability of the material has a critical role in determining surface repellency. From the perspective of materials design, lowering surface free energy and tailoring surface topographies are sometimes effective approaches to restrain the liquid contact and thus increase surface repellency. However, due to the complexity of the natural liquid/solid contact and solid/solid contact, the accretion mechanisms and controlling factors for surface repellency are highly dependent on specific matters of concern and the real application environments. When phase changes occur or multiple phases are involved, the influencing factors of surface accretion and repellency will be more complex. For example, superhydrophobicity is one of the popular research streams in icephobic surface design, but its effectiveness in icephobicity may be questioned under certain conditions. Further studies are required to develop new materials and surfaces that can offer better capability and applicability for different environments, as well as long-term durability in practical applications.

The research Interest of the topic“"New Insights in Wettability and Surface Repellency of Advanced Materials” includes, but is not limited to, the following: wettability, surface repellency, icephobic materials, antibacterial surfaces, slippery liquid-infused porous surfaces (SLIPS), and elastomer coatings. New material development for hybrid techniques of controlling surface accretion is also highly encouraged.
